# Effect of the allelic background on the phenotype of primary hyperoxaluria type I

**DOI:** 10.1097/MNH.0000000000001057

**Published:** 2024-12-06

**Authors:** Giorgia Mandrile, Barbara Cellini, Pietro Manuel Ferraro

**Affiliations:** a Genetic Unit and Thalassemia Center, San Luigi University Hospital, Orbassano; b Department of Medicine and Surgery, University of Perugia, Perugia; c Section of Nephrology, Department of Medicine, Università degli Studi di Verona, Verona, Italy

**Keywords:** kidney stones, pathogenic variant, primary hyperoxaluria, rare disorder

## Abstract

**Purpose of review:**

Primary hyperoxaluria type 1 (PH1) is an autosomal recessive disorder of hepatic glyoxylate metabolism leading to nephrolithiasis and kidney failure. PH1 is caused by mutations on the *AGXT* gene encoding alanine:glyoxylate aminotransferase (AGT). The *AGXT* gene has two haplotypes, the major (Ma) and the minor (mi) alleles. This review summarizes the role of the minor allele on the molecular pathogenesis and the clinical manifestations of PH1.

**Recent findings:**

PH1 shows high genetic variability and significant interindividual variability. Although the minor haplotype is not pathogenic on its own, it may be crucial for the pathogenicity of some mutations or amplify the effect of others, thus affecting both symptoms and responsiveness to Vitamin B6, the only pharmacological treatment effective in a selected group of PH1 patients.

**Summary:**

In the last years, new drugs based on RNA-interference are available for patients nonresponsive to Vitamin B6, but no specific biomarkers are available to predict disease course and severity. Therefore, a clinical assessment of PH1 taking into account molecular analysis of the mutations and the allelic background and the possible synergism among polymorphic and pathogenic variants should be encouraged to promote approaches of personalized medicine that improve the management of available resources.

## INTRODUCTION

**FIGURE 1:**
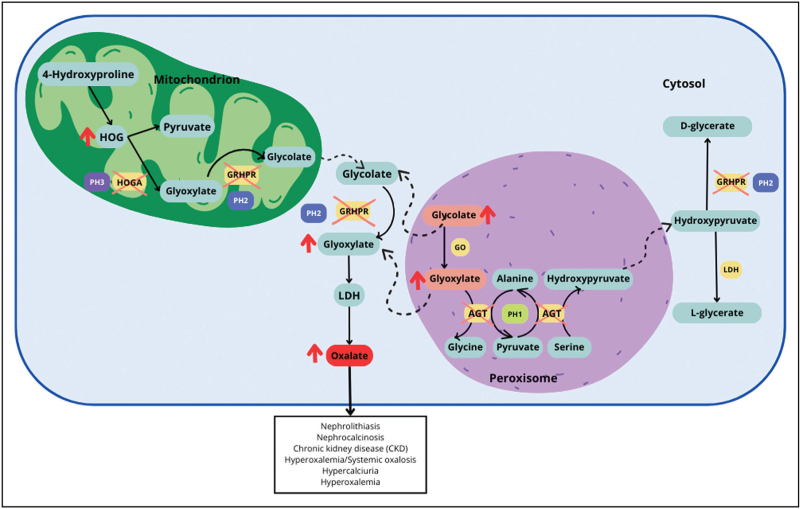
Summary of primary hyperoxaluria pathogenic pathways. AGT, alanine:glyoxylate aminotransferase; GO, glycolate oxidase; GRHPR, glyoxylate reductase/hydroxypyruvate reductase; HOG, 4-hydroxy-2-oxoglutarate; HOGA, 4-hydroxy-2-oxoglutarate aldolase; LDH, lactate dehydrogenase; PH1, primary hyperoxaluria type 1; PH2, primary hyperoxaluria type 2; PH3, primary hyperoxaluria type 3.

Primary hyperoxalurias (PHs) are autosomal recessive disorders of hepatic glyoxylate metabolism leading to abnormal endogenous oxalate synthesis and urinary excretion, which in turn causes nephrolithiasis, nephrocalcinosis and kidney failure with systemic oxalate accumulation [1^▪▪^,2^▪^]. Three forms of PH are currently known named types 1, 2 and 3, caused by mutations on the *AGXT*, *GRHPR* and *HOGA1* gene, respectively. PH1 is the most common and the most severe form of disease, accounting for 80% of all PH patients [1^▪▪^]. PH2 affects about 10% of cases and can lead to kidney impairment in about 50% of patients, with 25% of them reaching kidney failure [3^▪^]. PH3 accounts for 8–10% of cases and is the mildest form, characterized by recurrent nephrolithiasis, particularly in the first decade of life, with kidney function usually preserved [4^▪^] (Fig. 1). 

**Box 1:**
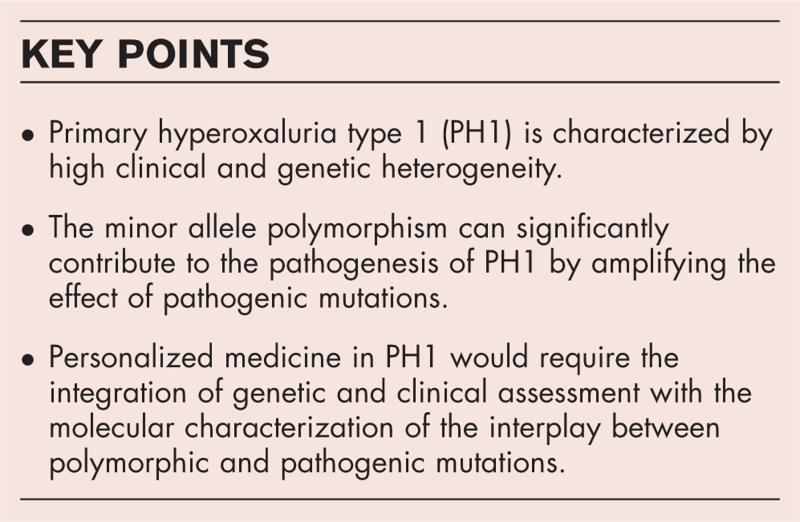
no caption available

In PH1, pathogenic mutations in the *AGXT* gene cause a deficit of alanine:glyoxylate aminotransferase (AGT), which catalyzes the transamination of glyoxylate to glycine in liver peroxisomes [5–7,8^▪^]. The AGT deficit prevents glyoxylate detoxification while allowing its oxidation to oxalate, leading to calcium oxalate accumulation and precipitation as stones in the kidneys and urinary tract [9^▪^,^10^]. More than 400 *AGXT* pathogenic variations have been described, with different frequency on the basis of the ethnic background [11–14].

Genetic testing is the gold-standard for the diagnosis [1^▪▪^,2^▪^,9^▪^,^15^] and should be performed as soon as possible in order to guarantee family counseling and adequate treatment, since in PH1 a strong genotype-phenotype correlation has been demonstrated [16^▪^,17^▪^]. Traditionally, vitamin B6 (pyridoxine – VB6) supplementation was the only pharmacological treatment, effective in a selected group of PH1 patients, while the only curative option was kidney-liver transplant [18,19]. In the last years, the management of PH1 has been significantly boosted by the availability of new biological drugs based on RNA-interference [20^▪^,^21^,22^▪^,^23^–^25^] for patients non responsive to VB6 [1^▪▪^,^26^]. It has therefore become increasingly important for clinicians to be aware of the disease as well as of the benefits and limitations of each therapeutic strategy based on the genetic background of each patient, in order to favor the transition to personalized medicine and a better management of the available resources.

## CLINICAL VARIABILITY IN PRIMARY HYPEROXALURIA TYPE 1

In PH1 patients, the intratubular and interstitial calcium oxalate deposits caused by the increased hepatic production of oxalate, along with chronic tubulo-interstitial inflammation and urinary tract obstruction, result in kidney failure in over 70% of patients [1^▪▪^,8^▪^]. Because of the progressive decline in glomerular filtration rate, plasma oxalate levels increase and, when supersaturation threshold is reached (30 μmol/l), oxalate accumulates and precipitates as calcium oxalate deposits in several organs and tissues, determining systemic oxalosis. This life-threatening condition is characterized by the involvement of skin, vessels, eyes, myocardium, heart conduction system, bones, bone marrow and central nervous system, substantially increasing the disease burden due to high morbidity and mortality [27,28]. Clinically, PH1 is usually characterized by a very early onset of systemic oxalosis (at age < 1 year), associated with failure to thrive, vomiting, bilateral nephrocalcinosis, oligo-anuria and rapid progression to kidney failure [1^▪▪^,^29^], occurring in 50% of cases before the age of 25 years [9^▪^]. In addition, a recent study showed that nephrocalcinosis at time of PH1 diagnosis is associated with a 3-fold increased risk of kidney failure [30].

Therefore, a timely diagnosis is necessary. PH1 is diagnosed through a molecular genetic testing that identifies biallelic pathogenic mutations on the *AGXT* gene. Diagnosis usually occurs during adolescence or adulthood, although the disease can manifest at any age and, sometimes, neglected symptoms may be present from infancy [29,31]. Indeed, a substantial number of patients receive their first diagnosis after kidney failure or kidney transplantation [31]. Achieving a definitive diagnosis can represent an arduous challenge particularly for adult patients, as demonstrated by the diagnostic delays reported in several papers [11,32,33]. Biochemical assessment through urinary and/or plasma oxalate level determination is also important to support PH1 diagnosis, as well as to measure the effectiveness of the therapeutic strategy adopted by the physician [1^▪▪^,2^▪^].

Nonetheless, PH1 is characterized by significant interindividual variability, with clinical phenotypes ranging from mild symptoms to extremely severe manifestations. Different studies demonstrated a strong connection between genotype, PH1 phenotype and treatment effectiveness [9^▪^,16^▪^,17^▪^]. Therefore, comprehensive genetic testing is mandatory. With the advent of next generation sequencing, PH1 molecular diagnosis has become easily available, but laboratories should be trained in taking into account also the presence of rare deletional variants [14] and in reporting the allelic background. Indeed, the *AGXT* gene has two common haplotypes, the major (Ma) and the minor (mi) alleles, which show a frequency of 80% and 20%, respectively, in white population. The minor allele, which is characterized by the two amino acid substitutions p.Pro11Leu/p.Ile340Met, is not pathogenic on its own, but it may amplify the effects of different common PH1-associated mutations or even determine the responsiveness to VB6 [34^▪^,^35^].

In particular, patients with homozygous p.Gly170Arg or p.Phe152Ile mutations, in combination with the minor allele polymorphism are more likely to benefit from VB6 therapy, with a substantial reduction, and in some cases normalization, of urinary oxalate (UOx) levels [36,37^▪^]. Moreover, these patients typically experience kidney failure at a significantly older median age compared to other PH1 patients with VB6-unresponsive mutations [1^▪▪^,^36^,37^▪^]. On the other hand, the p.Trp108Arg variant on the minor allele shows strongly decreased catalytic activity and coenzyme binding affinity, suggesting a poor response to VB6 supplementation [11,17^▪^]. Therefore, it is clear that an accurate analysis of the variants and their interaction with the haplotype may help to define the appropriate management of these patients.

The first line approaches in PH1 treatment include patient hyperhydration and urine alkalinization, both aiming at reducing the risk of calcium oxalate precipitation. The administration of VB6 is then recommended in all PH1 patients, along with the analysis of urinary oxalate excretion to assess responsiveness [1^▪▪^,2^▪^,^38^,^39^]. Because the potential VB6 effect on several mutations is still not clear, all patients should be offered a VB6 trial in order to determine the clinical response [1^▪▪^,2^▪^]. From a trial performed in 12 patients [30] and other observations [17^▪^,^40^,^41^], it is expected that approximately about 10–20% of PH1 patients completely normalize their UOx excretion in response to VB6, 30% may experience a partial response, and 50–60% would be refractory, at least in Western Europe and the USA where VB6-sensitive mutations are more prevalent than in other parts of the world [37^▪^]. Patients unresponsive to VB6 usually underwent combined or sequential liver–kidney transplant, as it represented the only curative therapy to restore normal AGT activity in the liver and prevent oxalate burden [42]. The recently approved biological drugs for PH1, Lumasiran and Nedosiran, have proven very effective in reducing urinary oxalate excretion, so that their use must be considered in patients unresponsive to VB6 [21,24]. However, further studies are necessary to draw solid conclusions about their role in the clinical outcome as well as the possibility to avoid liver transplantation [1^▪▪^,^43^,^44^]. In addition, the new therapies have high costs and further highlight the need of additional efforts to better understand PH1 pathophysiology and implement new tools to predict disease course and response to treatment.

## ROLE OF THE GENETIC BACKGROUND ON THE CLINICAL AND MOLECULAR PHENOTYPE OF PRIMARY HYPEROXALURIA TYPE 1

One of the challenging questions in PH1 is the high genetic variability of the disease, which is not only related to the high number of pathogenic mutations associated with the disease, but also to the presence of two polymorphic haplotypes [9^▪^]. In fact, a vast number of pathogenic mutations are missense and they can lead to the functional deficit of AGT through a variety of mechanisms [19,45,46]. It is known that only a minority of the mutations involve catalytic residues, while most of them affect the folding of the protein and reduce its fitness, so that the intrinsic catalytic ability is preserved, but the enzyme levels are so low that they do not allow proper liver glyoxylate detoxification [19,46–48]. The complexity is further exacerbated by presence of two polymorphic forms of AGT in humans; the minor allele can worsen the effect of co-inherited specific pathogenic mutations, that are pathogenic only if inherited on the mi background, while other variants give rise to a more severe protein defect when associated with the minor allele as compared with the major one [47,49,50^▪^,51^▪^].

Human AGT is a dimer and catalyzes the transamination of L-alanine to pyruvate and glyoxylate to glycine using as coenzyme pyridoxal 5-phosphate (PLP), a derivative of VB6 bound to each monomer through a Schiff base with Lys209 [52]. The two polymorphic forms of the protein (Ma and mi) do not show significant differences in terms of catalytic activity, but are endowed with a different thermodynamic stability [50^▪^,^53^,^54^], which results from an increased structural flexibility [50^▪^,55^▪^] and translates into an increased tendency to aggregation that reduces protein fitness in vitro and cells [54,56,57]. All these perturbations are mainly driven by the p.Pro11Leu substitution, while the p.Ile340Met mutation appears to exert a stabilizing role [50^▪^,^53^]. In addition, the Pro11-to-Leu substitution favors the mistargeting of AGT to mitochondria, where the enzyme cannot exert its metabolic role in glyoxylate detoxification [51^▪^]. However, AGT mistargeting only occurs as a synergistic effect of the polymorphic change with some pathogenic mutations, namely p.G170Arg, which redirects nearly all the enzyme to mitochondria and therefore causes the disease only in the presence of the minor allele polymorphism [58^▪^]. Notably VB6 supplementation is effective in patients with the p.Gly170Arg-mi variant, by increasing the net AGT expression, catalytic activity, and peroxisomal targeting [40,58^▪^]. A similar mechanism explains the synergy minor allele/pathogenic mutation for the p.Phe152Ile and p.Gly47Arg mutations, which interfere with the coenzyme binding causing a destabilization and consequent aggregation of AGT in the apo-form, and respond to VB6 that shifts the protein to the holo form, thus preventing aggregation and mistargeting to mitochondria [9^▪^,59^▪^,^60^].

Based on these reports, an increasing number of *AGXT* mutations were studied to allow a better comprehension of the molecular mechanisms of the disease, improve diagnosis, and offer targeted treatments to patients. In this regard, it has been found that some mutations are pathogenic on both allelic backgrounds, but the severity of their effects and/or the response to VB6 is different based on the absence or presence of the minor allele polymorphism. This is the case of the mutation p.Ile56Arg [49,61], whose effect on AGT stability increases based on the residue present at position 11 (Arg>Leu>Pro). Interestingly, this mutation shows a response to VB6 only when associated with the major allele (i.e. proline at position 11). This could be related to the synergic destabilization of the N-terminus of AGT caused by the concomitant presence of mutations at Ile56 and Pro11, that overcomes the rescuing effects of VB6, and correlates with the absence of clinical response observed when the mutation is inherited on the minor allele [61]. Similar results have been obtained for the p.Gly41Arg mutation. Indeed, when present on the minor haplotype, this mutation leads to intraperoxisomal aggregation and partial mistargeting, without a significant effect of VB6 on activity and subcellular localization [62^▪^], in line with a severe clinical phenotype [17^▪^]. However, when the mutation is present on the major haplotype, the phenotype is milder as compared with the same mutation on the minor allele, and data obtained in a cellular model of disease clearly show responsiveness to VB6 [63^▪^]. Accordingly, a case of significant response (UOx reduction over 50%) has been reported in a p.Gly41Arg homozygote bearing the mutation on the major haplotype, thus supporting further speculations on the effect of the haplotype on the phenotypic expression of this mutation [41].

From these examples it follows that a correct assessment of PH1 would greatly benefit from a deep molecular analysis of the effect of each identified mutation, as well as on the study of its possible synergism with polymorphic variants.

## THE TRANSLATIONAL POTENTIAL OF THE MOLECULAR DIAGNOSIS IN PRIMARY HYPEROXALURIA TYPE 1

Thanks to the availability of specific in vitro and in vivo models, the molecular properties of many disease-causing *AGXT* variants are currently available [64]. Nonetheless, a number of mutations still remain uncharacterized and no specific biomarkers are available to predict disease course and severity, in particular for what concerns the progression in the decline of kidney functions [1^▪▪^,^26^,^65^]. The advent of next-generation sequencing and the consequent use of whole exome sequencing and whole genome sequencing has greatly improved the diagnosis of all rare diseases including PH1 [9^▪^,^66^]. These approaches give the opportunity to evaluate the clinical phenotype not only focusing on the specific pathogenic mutation responsible for the AGT deficit, but also considering the co-inheritance of polymorphic mutations that can modulate the severity of the symptoms and/or determine the response to the available treatments.

Not only in case of genetic variants of uncertain significance, but also for rarer pathogenic variations, requesting support from a reference center of expertise can offer a deepest characterization and, therefore, ensure better patient management. In general, a precise definition not only of the pathogenicity of the *AGXT* variant but also of the allelic background can facilitate targeted therapy and the adequate administration of novel therapies, in order to personalize the patient's treatment and to optimize the resources utilization.

## CONCLUSION

**FIGURE 2:**
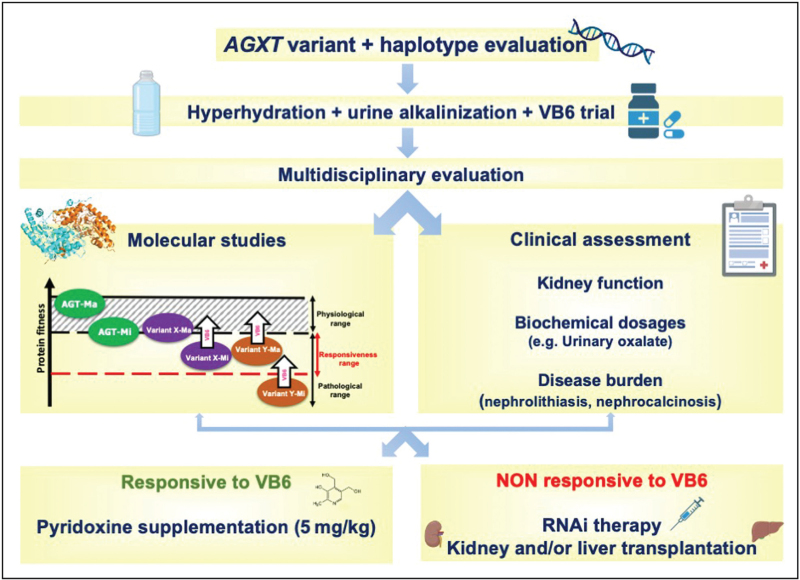
Schematic representation of the interplay between clinical and molecular assessment for treatment personalization in PH1. After molecular diagnosis and first-line treatment with hyperhydration and urine alkalinization, the subsequent clinical management should be tailored on the basis of the patient's clinical and molecular assessment (e.g. evaluation of the interplay between AGXT mutation and allelic haplotype). VB6, Vitamin B6. See text for details.

Going forward, we can expect a transition to an integrated evaluation of clinical data, genetic background and molecular studies in order to 1) improve the diagnosis of the disease, by also including the functional analysis of newly-identified variants or variants of unknown significance using available models; 2) implement integrated platforms of precision medicine, which starts from the knowledge of the pathogenic mechanisms to give insights into the best treatment to optimize the risk/benefit ratio for each patient (Fig. 2).

## Acknowledgements

*We thank Noemi Giordano for her help in pictures drawing.*

### Financial support and sponsorship

*None.*

### Conflicts of interest

*B.C. has received a grant from Dicerna Pharmaceuticals, a Novo-Nordisk subsidiary, Lexington, MA, USA. PMF received consultant fees and grant/other support from Allena Pharmaceuticals, Alnylam, Amgen, AstraZeneca, Bayer, Gilead, Novo Nordisk, Otsuka Pharmaceuticals, Rocchetta, Vifor Fresenius, and royalties as an author for UpToDate. GM received consultant fees and grant/other support from Alnylam and Novo Nordisk.*

## REFERENCES AND RECOMMENDED READING

Papers of particular interest, published within the annual period of review, have been highlighted as:

▪ of special interest
▪▪ of outstanding interest
